# Effectiveness of a multidimensional intervention program in improving occupational musculoskeletal disorders among intensive care unit nurses: a cluster-controlled trial with follow-up at 3 and 6 months

**DOI:** 10.1186/s12912-021-00561-y

**Published:** 2021-03-20

**Authors:** Shuai Yang, Li Li, Liqian Wang, Jiaqi Zeng, Bin Yan, Yinglan Li

**Affiliations:** 1 Nursing School, Jinan University, Guangzhou City, Guangdong Province China; 2 Xiangya Nursing School, Central South University, 172 Tongzipo Road, Yuelu District, Changsha, Hunan China; 3 Xiangya Hospital of Central South University, Changsha City, Hunan Province China; 4 The Third Xiangya Hospital of Central South University, Changsha City, Hunan Province China

**Keywords:** Musculoskeletal disorder, Intensive care unit, Nurses, Intervention study

## Abstract

**Background:**

Intensive care unit (ICU) nurses are at high risk for work-related musculoskeletal disorders (WRMDs). Data on occupational injuries indicate the significance of WRMDs among ICU nurses. Intervention programs have previously been developed to reduce WRMDs, but different intervention methods need to be adopted for different groups of people. This study aimed to evaluate the effectiveness of a multidimensional intervention program to prevent and reduce WRMDs in ICU nurses.

**Methods:**

This study was designed as a two-armed cluster-controlled trial with an intervention group and a control group. The clusters were independent hospital ICUs, and the participants consisted of registered nurses in China. By cluster random sampling, 89 nurses from two ICUs were assigned to the intervention group, and 101 nurses from two other ICUs were assigned to the control group. A multidimensional intervention program based on previous studies was designed. This program combined improving risk perception, health behavior training, and promoting a safe working environment. The multidimensional intervention program was implemented in the intervention group, whereas routine specialist training was implemented in the control group. Baseline and follow-up (3 and 6 months) data were collected using self-reported online questionnaires. The primary outcome was the report rate of WRMDs in the past 7 days. Secondary outcomes were risk perception, application of health behavior, and perception of a safe working environment. The data were statistically analyzed using SPSS 19.0.

**Results:**

A total of 190 nurses provided three recorded outcome measurements (intervention group, *N* = 89 (94.68%); control group, *N* = 101 (94.39%)). After 6 months, the intervention group experienced significant improvement relative to the control group in the report rate of WRMDs in the past 7 days (OR = 1.953, *p* = 0.037), risk perception (OR = 0.517, *p* < 0.001), application of health behavior (OR = 0.025, *p* < 0.001), and perception of a safe working environment (OR = 1.637, *p* = 0.024).

**Conclusion:**

The multidimensional intervention program was superior to routine specialist training in preventing the occurrence of WRMDs in ICU nurses. WRMD training should include multifaceted approaches and pay increased attention to specific department functions.

## Background

Work-related musculoskeletal disorders (WRMDs) are injuries to the musculoskeletal system that result from exposure to the work environment [1]. For nurses, WRMDs are major occupational health problems. The 12-month prevalence of WRMDs among nurses worldwide ranges from 40 to 85%; the difference is attributed to variations in the national culture, medical environment, and nature of the job [2–5]. The resulting disorders can lower the quality of life and increase the number of sick days of the nursing staff [6, 7]. In China, a large number of nurses often work with disorders because of a shortage of nursing staff and a lack of awareness of the cumulative damage of WRMDs [8]. In a survey of WRMDs among Chinese nurses, the 12-month prevalence of WRMDs was 77.43%, but only 9.39% of nurses took a sick leave due to WRMDs [9].

An intensive care unit (ICU) is a place that provides rescue and treatment for critically ill patients. Patients in the ICU are often immobile with severe physical weakness and a limited ability to care for themselves. Nurses are required to complete a large amount of professional treatment, rescue, and care every day, rendering them vulnerable to developing WRMDs [10–12]. Our recent cross-sectional survey showed that WRMDs have a prevalence of 97.1% among ICU nurses in Chinese tertiary hospitals, which is close to that (95–98%) among ICU nurses surveyed by Zhang et al. [13]. WRMDs negatively affect the well-being and work of ICU nurses, and this effect has been shown to be significant [14]. Chen and Li have demonstrated that 28.9% of ICU nurses in China filed a leave of absence or requested a transfer to another unit because of lower back pain [15]. Therefore, WRMD prevention is important for the health of nurses and the stability of ICU teams.

Many single intervention programs have been implemented to reduce the risk of WRMDs, including patient handling and mobility programs [16], ergonomic intervention [17, 18], work-related psychosocial coaching [19], health promotion and health protection intervention [20, 21], exercise, physiotherapy [22, 23], and so on. However, a systematic review of prevention and reduction of WRMDs among nurses indicates that evidence is limited for each intervention type [24]. Tullar et al. have conducted a systematic review of interventions for musculoskeletal injuries in health care workers and reached a similar conclusion—that is, training alone or exercise alone exerts no effect on musculoskeletal health [25]. Therefore, a broader perspective is needed for the prevention of WRMDs. Occupational risk factors for WRMDs are not independent. The occurrence of WRMDs in nurses may be influenced by physical factors as well as environmental factors, organizational factors, and preexisting WRMD symptoms.

Some studies have explored the combination of two or more single-factor intervention programs for WRMDs into a multidisciplinary intervention program, which may be preferable [26, 27]. However, no standards have been set for a successful multidimensional intervention program to prevent WRMDs, and relevant evidence is limited [24]. Moreover, research is rarely reported on multidimensional intervention programs that provide a specialized program based on the needs of a specific group of nurses and focus on the factors affecting WRMDs in that group. It is possible that a multidimensional intervention program can be customized based on work characteristics, ergonomic equipment available in the workplace, medical environment characteristics, and national cultural characteristics. This type of program can be more closely targeted to prevent injuries and the recurrence of WRMDs. This study aimed to evaluate the effectiveness of such a multidimensional intervention program.

More specifically, this study intended to evaluate the effectiveness of a multidimensional intervention program in reducing WRMD symptoms among ICU nurses in China. The multidimensional intervention program consisted of three components: improvement of risk perception, health behavior training, and promotion of a safe working environment. Risk perception has been proposed as a determinant for preventive health behaviors in a number of behavioral theories [28]. Adequate perception of WRMDs can motivate the adoption of safe work behaviors [29, 30]. In addition, health behavior programs and physical factors are among the main risk factors for WRMDs that have shown potential in numerous intervention studies [31, 32]. Environmental safety in hospitals is also one of the risk factors affecting the occurrence of WRMDs. A positive perception of working environment safety can effectively reduce the risk of occupational exposure [33]. Therefore, the present study provides evidence for multidimensional intervention programs for the prevention of WRMDs.

## Methods

### Study design

This study was designed as a two-armed cluster-controlled trial with an intervention group and a control group, where the clusters were independent hospital ICUs in China, and the participants consisted of registered nurses in clinical practice. In China, all nursing directors in public hospitals are required to organize an occupational health training program, including comprehensive training and routine specialist training [34]. Comprehensive training involves uniform training of basic theories and skills for nurses by the hospital. Routine specialist training is organized by the head nurse and is conducted in the form of lectures once or twice a year, depending on the occupational risks of the unit. Routine specialist training includes special disease care as well as information and training on occupational health risks [35]. After baseline data were collected (as described below), the participating ICUs were randomly divided into the intervention group and the control group. A two-month multidimensional training program was implemented in the intervention group, whereas only routine specialist training was implemented in the control group. Stratified randomization was not possible because the towns where the participants were located were far apart, and the potential propensity to communicate with each other was present, considering that the participants were working in the same hospital.

This study followed the criteria for the development and evaluation of training interventions for healthcare professions recommended by the Equator Network [36]. No other specific risks were associated with participating in this program other than those associated with the adoption of preventive actions. The Research Ethics Committee of Xiangya Nursing School of Central South University approved this study (2017025).

### Setting

The programs for the intervention group and the control group were implemented in ICU rooms for the convenience of the nurses. All ICUs participating in this study had similar levels of health care complexity. Each nurse was responsible for an average of 2–3 patients on a shift and had received routine specialist training with similar professional training methods and content.

### Recruitment of ICUs

The ICUs had to meet the following criteria to ensure the selection of appropriate clusters and the implementation of the intervention: (1) the ICU was located in a tertiary public hospital; (2) the ICU admitted mixed cases (i.e., ICUs with critically ill patients could have been transferred from any department); (3) commitment and explicit interest to implement the programs and evaluation were shown by the hospital nursing directors, head nurses, and nurses; and (4) the ICU was exposed to significant risks of WRMDs, as assessed by the previous survey (prevalence of WRMDs > 90%) [14].

The units were recruited from ICUs in Hunan Province, China, that participated in the previous cross-sectional survey. Under the aforementioned criteria, four mixed ICUs in four tertiary public hospitals were recruited. An independent researcher randomly assigned the four ICUs to the intervention group (two ICUs) or to the control group (two ICUs) by using the random grouping function in Excel.

### Recruitment of participants

During recruitment, the purpose and methods of the intervention study were explained orally to the participants in each cluster before they were assigned to their respective group: the intervention group or the control group. Informed consent and the baseline questionnaire were obtained from each participating ICU nurse. After the questionnaires were completed and returned, the 4 units were randomized, and the participants were informed of whether they were in the intervention group or the control group. Subsequently, the intervention started. Follow-up questionnaires were administered at 3 and 6 months after the start of the intervention.

### Eligibility criteria of participants

To be included in this study, the participants needed to be registered nurses, including nurses who were on sick leave and those who were engaged in patient care daily, and wanted to volunteer. Nurses who were pregnant and performed only administrative work were excluded from this study.

### Intervention

Our previous cross-sectional survey identified the following risk factors for WRMDs among ICU nurses in Chinese tertiary hospitals: risk perception, physical factors (frequency of handling patients and physical workload), psychosocial factors (job stress), and workplace environmental factors [14]. Accordingly, a personalized and multidimensional intervention program was designed to reduce WRMD symptoms, as reported by Chinese ICU nurses. The multidimensional intervention program consisted of three components: improvement of risk perception, health behavior training, and promotion of a safe working environment.

**Fig. 1:**
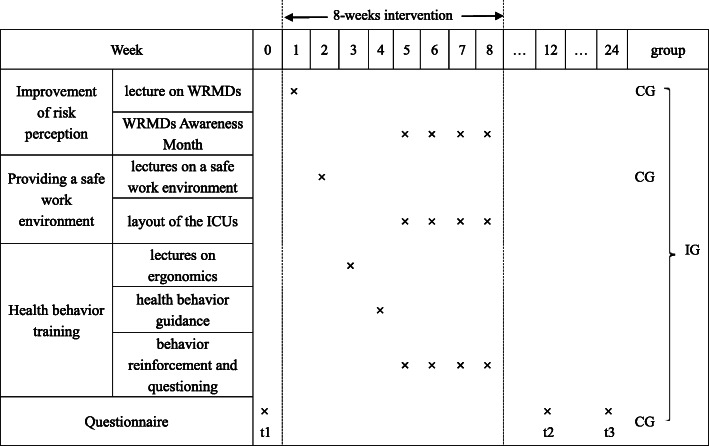
Intervention process. WRMDs = work-related musculoskeletal disorders; ICUs = intensive care units; CG = control group; IG = intervention group; t1–t3 = measurement points

The multidimensional intervention program was implemented in the intervention group. The control group received routine specialist training on WRMDs, including two lectures on WRMDs and safe working environments. The schedule of the specific interventions is presented in Fig. 1. A working group was organized for each hospital ICU. The intervention group included a head nurse, an ergonomics specialist, an orthopedist, a nurse representative, and a researcher responsible for the development and implementation of the intervention. The control group included a head nurse, a nurse representative, and a researcher responsible for routine specialist training on WRMDs.

#### Improvement of risk perception of WRMDs

Risk perception plays an important role in preventing occupational risks and can be used as an incentive to promote safe work behaviors [37]. The measures used to improve risk perception were as follows: (1) *A lecture on WRMDs consisting of a 40-min course that had been determined by the working group.* The lecture covered the type, symptoms, epidemiology, risk factors, and consequences of WRMDs and was presented by clinical nursing experts with considerable years of experience in WRMDs. (2) *WRMD Awareness Month.* The Health Belief Model holds that individual behavior is influenced by cues that motivate people to change their behavior, such as media reports, advice from others, and reminder brochures [28, 29]. Weeks 5 to 8 of the intervention program were assigned as the WRMD Awareness Month. The working group distributed brochures on WRMDs to nurses. The brochures covered the concepts of WRMDs, influencing factors, protective exercises, and application of the principles of ergonomics. A competition on WRMD knowledge and skills was held during the awareness month. It focused on strengthening the knowledge and skills of nurses in order to prevent WRMDs. Nurses with excellent grades were rewarded.

#### Health behavior training

Some studies have shown that physical interventions, such as the use of ergonomic aids, training in patient handling, and physical activity, positively affect the reduction of musculoskeletal injuries and pain among nurses [24]. The health behavior training in our study included the following: (1) *A 40-min lecture on ergonomics*. An ergonomic expert introduced the concept of ergonomics, research content, and related principles and applications of nursing practice. (2) *Health behavior guidance*. A science-based guidance plan was developed by the working group and implemented in the intervention group. Specifically, the behavior guide included suggestions on how to (i) move and carry bedridden patients, (ii) use slides when moving an awake patient from the bed to a wheelchair as well as carrying and moving objects, (iii) lift items, (iv) walk, squat, and turn around at work; (v) adopt the correct sitting posture; (vi) adjust the chair height for fit and comfort; and (vii) perform physical exercises. Each procedure was demonstrated and guided by ergonomics and clinical nursing experts. (3) *Health behavior reinforcement and questioning*. The Transtheoretical Model of Change claims that changes in human behavior should undergo consolidation and recurrence [38]. Thus, a weekly session starting at week 5 was organized by the working group to reinforce the health behavior of nurses and to solve problems. At the meeting, the nurses would review correct behaviors via scenario simulation and group discussion.

#### Providing a safe work environment

Poor perception of a safe working environment represents a stressor that may increase the number of WRMDs reported by employees [39]. Based on the inspection results and the recommendations by the ergonomic expert and the clinical nurse expert, the working group proposed and implemented the following: (1) *Lectures on a safe working environment, with each session lasting 40 min.* A clinical nursing expert introduced management support for working safety, barriers to work safety, safety awareness training, teamwork, and communication. (2) *An improvement plan for an ICU layout.* Work chairs (5–7) were replaced with height-adjustable chairs for nurses of different heights, a step stool (30 cm high) was provided to help nurses reach for items at a higher location, and slides (89 cm × 50 cm) were purchased to assist nurses when transferring patients from the bed to a wheelchair. At the same time, we ensured that every nurse mastered how to use the slide.

#### Routine specialist training in WRMDs

The control group received only routine specialist training, including: (1) lectures on WRMDs and a safe working environment, consisting of two 40-min sessions, which were consistent with the content of the intervention group. The training is updated yearly to meet the requirements for unit development.

Finally, all components of the intervention group and the control group required a leader to integrate, coordinate, and lead the research. The tasks involved communication, organizing meetings, and facilitating the implementation of the intervention.

### Measurement

Data were collected using self-reported online questionnaires. The online questionnaire was comprised of two parts. The first part consisted of informed consent. If nurses were willing to participate in the study, they completed the succeeding questionnaire, but if not, the nurse closed the application. The second part was the questionnaire itself. Baseline demographic information was collected, including age, gender, height, weight, marital status, job title, education, and number of years working in the ICU. Baseline and follow-up primary and secondary outcomes were collected, with follow-up conducted at 3 and 6 months.

This online questionnaire was developed using the Chinese-based questionnaire software Sojump (Sojump, Hu Xiao, China). The software generated an online link and a two-dimensional image code. By clicking on the link or scanning the two-dimensional image code, the nurses could enter and complete the questionnaire via the WeChat application on a mobile device. To prevent incomplete and duplicate data, the questionnaire contained mandatory fields and limited submission to one online questionnaire for every WeChat account. All respondents who completed the questionnaire received ¥5.0 (about US$0.71) as an incentive via the WeChat mobile payment red envelope function.

All sample data were exported from the Sojump software to SPSS 19.0 and were double-checked to identify inconsistencies and errors. Data for incomplete studies were not used for statistical analysis.

### Primary outcome

#### Report rate of WRMDs in the past 7 days

The Chinese version of the Nordic Musculoskeletal Questionnaire [40] was used to measure self-perceived symptoms of WRMDs in nine regions of the body during the last year and the past 7 days. A diagram of the body was included to allow nurses to identify the affected areas. No checkmarks or multiple checkmarks were allowed. Baseline and follow-up (3 and 6 months) data were collected.

### Secondary outcomes

#### Risk perception

Risk perception was assessed using the Chinese version of the Risk Perception of Musculoskeletal Injury developed by S. J. Lee et al. (2013) [41]. This tool was translated from English to Chinese by the researcher, with the permission of the author. The respondents estimated the risk of WRMDs as perceived by themselves or by other nurses in their respective units. A six-point Likert scale from 1 (extremely unlikely) to 6 (extremely likely) was used. The score was calculated as the mean of the eight items; the higher the score, the greater the WRMD risk perceived.

#### Application of health behavior

The application of health behavior was measured using the Nursing Physical Factors Evaluation Questionnaire, including the frequency of patient handling (6 items) and physical workload (9 items). This tool was designed by the author for this study. A five-point Likert-type scale from 0 (never) to 4 (very often) was used. The total score of the 15 items was considered as the final score; the higher the score, the greater the ergonomic risk.

#### Perception of a safe working environment

The Chinese version of the Hospital Safety Climate Questionnaire [42] was used to measure the level of awareness regarding workplace and environmental safety, which was slightly modified to fit the context of this study. All items were answered using a four-point Likert-type scale from 1 (strongly disagree) to 4 (strongly agree). The score was calculated as the sum of the items; the lower the score, the safer the environment was perceived to be. Baseline and follow-up (3 and 6 months) data were collected.

Additional details on the questionnaires are provided in the previous article on WRMDs by the author [14].

### Data analysis

The data were statistically analyzed using SPSS 19.0 (IBM, NY, USA). Descriptive statistical analysis was used to summarize the demographic characteristics of the participants. The Student’s *t-*test and the chi-squared test were used to determine whether a statistical difference existed between the intervention group and the control group at baseline. Analyses of the effectiveness of the primary outcome and the secondary outcomes were performed after intervention for 6 months by using a generalized estimation equation (GEE). The subject variable was the number of nurses, and the internal variable was the time point. The model type (linear regression or binary logistic regression) was selected based on the type of outcome indicator. We first analyzed the single-factor GEE, followed by the multifactor GEE, including the demographic factors affecting the outcomes.

## Results

### Study sample

A total of 201 nurses from four mixed ICUs in four hospitals were recruited from December 2017 to January 2018. These four hospitals were selected from the 20 tertiary hospitals in Hunan Province, China, that participated in the previous cross-sectional survey. From two ICUs, 94 nurses were assigned to the intervention group, and from the two remaining ICUs, 104 nurses were assigned to the control group by cluster random sampling. During the intervention, five nurses in the intervention group were lost to follow-up: two nurses resigned, two nurses transferred units, and one nurse took a leave of absence due to pregnancy. Meanwhile, six nurses in the control group were lost to follow-up: one nurse quit voluntarily, two nurses left for training, and three nurses took a leave of absence due to pregnancy. Ultimately, 190 nurses provided three recorded outcome measurements (intervention group, *N* = 89 [94.68%]; control group, *N* = 101 [94.39%]). No significant difference in loss to follow-up was found between the two groups (χ^2^ = 0.074, *p* = 0.862).

### Baseline data

At baseline, no significant differences in demographic characteristics, except for the educational level (Table 1), were found between the intervention group and the control group. Overall, male participants were underrepresented in both the intervention group and the control group. The average age was 28 years old for the participants in both groups. Most participants had completed a bachelor’s degree and had worked for less than 10 years. No significant difference in the prevalence of WRMDs was indicated between the two groups (χ^2^ = 0.710, *p* = 0.824).

**Table 1:** Demographic characteristics of intensive care unit nurses participating in this study (*N* = 190)

Variable	Control group	Intervention group	*p*-value
	Mean ± SD^a^ or n (%)	Mean ± SD^a^ or n (%)	
Age	28.83 ± 4.25	28.86 ± 4.13	0.956
Sex			
Male	19	18	0.333
Female	70	83	
BMI^b^			
< 18.5	14	16	0.901
18.5–23.9	58	65	
24.0–27.9	15	19	
≥ 28	2	1	
Marital Status			
Single	36	43	0.771
Married	53	58	
ICU^c^ employment			
1–2 years	10	14	0.191
3–5 years	32	26	
5–10 years	28	43	
> 10 years	19	18	
Job title			
Nurse	31	28	0.314
Senior nurse	39	47	
Supervisor nurse	19	26	
Education			
Junior college^d^	4	17	**0.008**
Bachelor	71	73	
Master/Doctoral	4	11	

^a^*SD* Standard deviation

^b^*BMI* Body Mass Index

^c^*ICU* Intensive care unit

^d^Junior college refers to full-time nurse training in a vocational and technical school

### Intervention effects

Gender affected the report rate of WRMDs in the past 7 days (*p* = 0.003). The GEE, including gender, indicated that the measures of the intervention group and the control group were statistically significant. The report rate of WRMDs in the past 7 days in the control group was 1.953 times that in the intervention group (OR = 1.953, *p* = 0.037). No interaction was observed between the measurement time and the group (*p* = 0.578). The results of the specific parameter estimations are listed in Table 2.

**Table 2:** Parameter estimation of the generalized estimation equation for the reported rate of WRMDs in the past 7 days

Variable	Single-factor analysis				Multiple-factor analysis			
	OR^a^	Upper	Lower	*p*-value	OR^a^	Lower	Upper	*p*-value
Age	1.042	0.976	1.112	0.220				
Sex								
Male	2.332	1.320	4.083	**0.003**	2.465	1.383	4.396	**0.002**
Female	1				1			
BMI^b^								
< 18.5	0.325	0.049	2.165	0.245				
18.5–23.9	0.256	0.041	1.590	0.144				
24.0–27.9	0.347	0.051	2.344	0.278				
≥ 28	1							
Marital Status								
Single	1.288	0.784	2.115	0.318				
Married	1							
ICU^c^ employment								
1–2 years	0.418	0.186	0.936	0.034				
3–5 years	1.528	0.736	3.173	0.255				
5–10 years	1.028	0.520	2.032	0.937				
> 10 years	1							
Job title								
Nurse	0.762	0.403	1.441	0.403				
Senior nurse	1.109	0.600	2.048	0.741				
Supervisor nurse	1							
Education								
Junior college	0.492	0.144	1.683	0.258				
Bachelor	0.765	0.265	2.212	0.621				
Master/Doctoral	1							
Group								
Group = 0 (Control group)					1.953	1.126	3.677	**0.037**
Group = 1 (Intervention group)					1			
Time								
Time = 0 (Baseline)					1.374	0.740	2.551	0.315
Time = 1(3 months)					1.129	0.766	1.642	0.526
Time = 2 (6 months)					1			
Group × time								
[Group = 0] × [Time = 0]					1.225	0.566	2.782	0.578
[Group = 0] × [Time = 1]					0.965	0.610	1.526	0.878
[Group = 0] × [Time = 2]					1			
[Group = 1] × [Time = 0]					1			
[Group = 1] × [Time = 1]					1			
[Group = 1] × [Time = 2]					1			

^a^*OR* Odds ratio

^b^*BMI* Body Mass Index

^c^*ICU* Intensive care unit

The GEE showed that the multidimensional intervention program improved the risk perception of WRMDs (OR = 0.517, *p* < 0.001) and health behavior application (OR = 0.025, *p* < 0.001), relative to that of the routine specialist training. Interactions between the measurement time and group were observed (*p* < 0.001). The results of specific parameter estimations are listed in Tables 3 and 4.

**Table 3:** Parameter estimation of the generalized estimation equation for the risk perception of WRMDs

Variable	Single-factor analysis				Multiple-factor analysis			
	OR^a^	Upper	Lower	*p*-value	OR^a^	Lower	Upper	*p*-value
Age	0.990	0.970	1.011	0.347				
Sex								
Male	0.987	0.831	1.172	0.879				
Female	1							
BMI^b^								
< 18.5	0.941	0.721	1.227	0.652				
18.5–23.9	0.966	0.760	1.228	0.780				
24.0–27.9	0.957	0.730	1.255	0.752				
≥ 28	1							
Marital Status								
Single	1.125	0.983	1.287	0.087				
Married	1							
ICU^c^ employment								
1–2 years	1.007	0.770	1.317	0.960				
3–5 years	1.028	0.853	1.240	0.769				
5–10 years	0.945	0.755	1.151	0.572				
> 10 years	1							
Job title								
Nurse	1.204	0.996	1.454	0.054				
Senior nurse	1.076	0.901	1.286	0.416				
Supervisor nurse	1							
Education								
Junior college	1.010	0.750	1.362	0.946				
Bachelor	1.143	0.946	1.380	0.167				
Master/Doctoral	1							
Group								
Group = (Control group)					0.517	0.424	1.629	**< 0.001**
Group = (Intervention group)					1			
Time								
Time = 0(Baseline)					0.441	0.372	0.523	**< 0.001**
Time = 1 (3 months)					1.076	0.924	1.252	0.346
Time = (6 months)					1			
Group × time								
[Group = 0] × [Time = 0]					1.740	1.345	2.252	**< 0.001**
[Group = 0] × [Time = 1]					1.305	1.038	1.640	**0.023**
[Group = 0] × [Time = 2]					1			
[Group = 1] × [Time = 0]					1			
[Group = 1] × [Time = 1]					1			
[Group = 1] × [Time = 2]					1			

^a^*OR* Odds ratio

^b^*BMI* Body Mass Index

^c^*ICU* Intensive care unit

**Table 4:** Parameter estimation of the generalized estimation equation for the application of health behavior

Variable	Single-factor analysis				Multiple-factor analysis			
	OR^a^	Upper	Lower	*p*-value	OR^a^	Lower	Upper	*p*-value
Age	1.008	0.845	1.202	0.928				
Sex								
Male	0.310	0.055	1.768	0.187				
Female	1							
BMI^b^								
< 18.5	8.790	1.276	36.639	0.033				
18.5–23.9	3.845	0.485	13.463	0.202				
24.0–27.9	5.393	0.689	28.236	0.095				
≥ 28	1							
Marital Status								
Single	3.116	0.729	13.323	0.125				
Married	1							
ICU^c^ employment								
1–2 years	0.769	0.071	8.367	0.829				
3–5 years	1.433	0.196	10.470	0.723				
5–10 years	0.329	0.049	2.218	0.254				
> 10 years	1							
Job title								
Nurse	3.326	0.514	21.514	0.207				
Senior nurse	0.773	0.125	4.774	0.781				
Supervisor nurse	1							
Education								
Junior college	0.435	0.016	11.867	0.622				
Bachelor	1.764	0.107	29.002	0.691				
Master/Doctoral	1							
Group								
Group = 0(Control group)					0.025	0.011	0.132	**< 0.001**
Group = 1 (Intervention gruop)					1			
Time								
Time = 0 (Baseline)					0.015	2.149	0.011	**< 0.001**
Time = 1 (3 months)					1.252	0.294	5.339	0.761
Time = 2 (6 months)					1			
Group × time								
[Group = 0] × [Time = 0]					23.932	21.197	46.157	**< 0.001**
[Group = 0] × [Time = 1]					3.818	0.341	42.757	0.277
[Group = 0] × [Time = 2]					1			
[Group = 1] × [Time = 0]					1			
[Group = 1] × [Time = 1]					1			
[Group = 1] × [Time = 2]					1			

^a^*OR* Odds ratio

^b^*BMI* Body Mass Index

^c^*ICU* Intensive care unit

Age and the length of ICU employment affected the perception of a safe working environment (*p* = 0.047 and *p* = 0.011 respectively). The GEE, including age and ICU employment, indicated that the measures of the intervention group and the control group were statistically significant. The perception of an unsafe working environment in the control group was 1.637 times that in the intervention group (OR = 1.637, *p* = 0.024). No interaction between the measurement time and the group was observed (*p* = 0.535). The results of specific parameter estimations are listed in Table 5.

**Table 5:** Parameter estimation of the generalized estimation equation for the perception of a safe working environment

Variable	Single-factor analysis				Multiple-factor analysis			
	OR^a^	Upper	Lower	*p*-value	OR^a^	Lower	Upper	*p*-value
Age	1.422	1.004	2.014	**0.047**	1.087	0.537	2.200	0.816
Sex								
Male	13.853	0.410	27.541	0.143				
Female	1							
BMI^b^								
< 18.5	0.002	4.233	1.215	0.058				
18.5–23.9	0.009	2.869	2.634	0.104				
24.0–27.9	0.017	3.710	7.986	0.195				
≥ 28	1							
Marital Status								
Single	0.266	0.016	4.496	0.359				
Married	1							
ICU^c^ employment								
1–2 years	0.016	0.005	11.583	0.366	0.278	5.037	53.207	0.771
3–5 years	0.784	0.001	0.320	**0.011**	0.013	1.352	11.950	0.211
5–10 years	1.095	0.028	21.904	0.886	1.081	0.011	29.596	0.974
> 10 years	1				1			
Job title								
Nurse	0.032	0.001	1.203	0.063				
Senior nurse	0.998	0.040	25.162	0.998				
Supervisor nurse	1							
Education								
Junior college	0.060	0.001	20.436	0.344				
Bachelor	1.036	0.027	39.181	0.985				
Master/Doctoral	1							
Group								
Group = 0 (Control group)					1.637	1.063	40.371	**0.024**
Group = 1 (Intervention group)					1			
Time								
Time = 0 (Baseline)					14.667	0.276	49.842	0.185
Time = 1 (3 months)					0.583	0.031	11.001	0.719
Time = 2 (6 months)					1			
Group × time								
[Group = 0] × [Time = 0]					0.222	0.002	25.915	0.535
[Group = 0] × [Time = 1]					0.396	0.008	19.679	0.642
[Group = 0] × [Time = 2]					1			
[Group = 1] × [Time = 0]					1			
[Group = 1] × [Time = 1]					1			
[Group = 1] × [Time = 2]					1			

^a^*OR* Odds ratio

^b^*BMI* Body Mass Index

^c^*ICU* Intensive care unit

## Discussion

The results of this study revealed that compared with routine specialist training, the multidimensional intervention program more positively influenced the rate of WRMDs reported in the past 7 days and exhibited stronger sustainability in reducing the rate. These findings are consistent with those previously reported that multidimensional interventions are preferable to single interventions in reducing musculoskeletal disorders or the risk of subsequent injuries in nurses [18, 43, 44]. The reason for the stronger positive effect may be that the formulation of the intervention program was based on a previous investigation of influencing factors. Thus, adopting a customized multidimensional intervention program for a specific population is recommended.

Considering that the study duration was less than 1 year, we did not investigate the annual prevalence of WRMDs. Current research shows that intervention programs used to effectively reduce the annual prevalence of musculoskeletal disorders in nurses are limited. The intervention program by Sharafkhani et al., which was based on the health belief model, effectively improved the health belief score of the nurses; however, no statistical difference was found in the prevalence of musculoskeletal disorders after intervention for 1 year [29]. Yan et al. used knowledge training and ergonomic intervention to reduce the occurrence of WRMDs among nurses in Xinjiang Province, China. The results showed that despite the reduction in the annual prevalence of WRMDs, the difference was not statistically significant [45]. The reason may be that no other staff was present to monitor the work behavior of the nurses or no benefit was perceived [46]. The development of a long-term approach to maintain the effectiveness of intervention programs needs to be addressed.

Research shows that psychosocial factors play a significant role in the emergence and persistence of musculoskeletal disorders [47, 48]. In the current study, nurses obtained relevant knowledge of WRMDs and their consequences through lectures, which effectively improved the risk perception of WRMDs. Training and lectures have been shown to effectively increase awareness of safety and self-protection [49] as well as to promote healthy practices among nurses [41]. The WRMD Awareness Month as a cue factor strengthened the belief in safe work behavior through brochure distribution and knowledge and skills competition [28, 29]. As part of the multidimensional intervention, improving risk perception fully considered the effects of psychological factors on behavior and promoted the adoption of safe working behavior among nurses through changes in attitude and beliefs.

Many studies have shown that health behavior training can effectively reduce the occurrence of WRMDs among nurses [50, 51]. In the current study, we adopted ergonomics as the theoretical guide, combined with the work characteristics of ICU nurses, and guided nurses to take a scientific posture to engage in clinical nursing work. The transtheoretical model, which suggests that changes in behavior occur in stages, proposes that behavior may return to its original state [52]. Thus, we adopted intensive training to consolidate the effects of behavior training. The results showed that health behavior training effectively increased the number of nurses adopting health behavior and improved the short-term rate of reported WRMDs. This result is consistent with the previous evidence that courses on patient transport can significantly decrease the rate of short-term physical disorders. However, no significant difference in the reduction of physical disorders was determined between the baseline data and the data after 2.5 years [53]. Therefore, we recommend regular health behavior training to prevent musculoskeletal disorders. The appropriate period may be 6 months to 1 year.

A slide is a small auxiliary device used to transport a patient or move a patient from the bed to a wheelchair. Some studies support the use of slides as a part of musculoskeletal injury prevention programs as they can reduce the risk of musculoskeletal injuries for nurses [54, 55]. However, the current study showed a considerably low usage rate of slides (once every 2 days) among the ICU nurses. The reason could be that most patients in the ICUs could complete basic treatment and examination in their bed, and the slide could be too slippery for certain patients.

Multidimensional interventions also effectively reduced the perception of an unsafe working environment among nurses. It has been reported that nurses with a higher perception of a safe working environment have lower occupational hazard exposure and a lower incidence of musculoskeletal disorders [33, 56]. Studies on interventions for a safe hospital environment are rarely conducted, which may be attributed to the relatively fixed working environment in ICUs as well as the financial and administrative support required to implement changes in the workplace [16]. Considering the aforementioned limitations, we focused on the scientific placement of objects and the use of low-cost assistive equipment. These changes to the work environment are visible and sustainable; in addition, they actively involve nurses, effectively reducing the perception of an unsafe working environment among nurses.

Throughout the intervention process, the ICU nurses easily cooperated in the intervention programs. Most people were willing to participate and insisted on completing the process. However, some limitations require caution in interpreting the findings. First, the educational levels of the intervention group and the control group at baseline exhibited a significant difference, but education exerted no effect on the outcomes. Thus, the multidimensional intervention program is preferred. Second, th study relied on the memory of the participants, which might have been influenced by information bias from errors of recall. Third, this study had a short time frame and did not investigate the annual prevalence of WRMDs, considering that no statistical difference in reducing the annual prevalence of WRMDs has been indicated in the current intervention studies. Therefore, we hope to develop a multidimensional and short-term intervention program as part of an annual routine specialist training program for nurses.

## Conclusion

For occupational health promotion, meticulous planning is essential in order to make interventions compatible with the daily work routine (e.g., shift work). Under these circumstances, the multidimensional intervention program seems applicable from time, financial, and organizational perspectives. Compared with routine specialist training alone, the multidimensional intervention program helped to reduce the short-term reported incidence rate of WRMDs, improve the nursing risk perception and health behavior application, and promote a safe working environment. Hospitals should acquire appropriate handling or transfer equipment to reduce the number of nurses carrying patients manually. Routine specialist training should include multifaceted approaches (lecture and health behavior training) and pay more attention to the specific department functions. In addition, we recommend conducting multidimensional interventions for WRMDs in routine specialist training annually to regularly monitor nursing practices and to enhance the risk awareness among nurses, rather than relying on a single intervention.

## Data Availability

The datasets used and/or analyzed during the current study are available from the corresponding author on reasonable request.

## Abbreviations

WRMDs: Work-related musculoskeletal disorders; ICU: Intensive care unit;
GEE: Generalized estimation equation
